# Exploring the role of country-level gender equality in the link between relationship status and perceived social support across 49 countries

**DOI:** 10.1038/s41598-024-52304-z

**Published:** 2024-01-29

**Authors:** Agnieszka E. Łyś, Katarzyna Adamczyk

**Affiliations:** Faculty of Psychology and Cognitive Sciences, Adam Mickiewicz University in Poznań, Poznań, Poland

**Keywords:** Psychology, Human behaviour

## Abstract

Past studies have demonstrated that higher perceived social support among coupled individuals and greater gender equality foster a more supportive social context. Less is known about how the link between relationship status and perceived social support may vary across countries that differ in gender equality. Employing the data collected from the COVIDiSTRESS I (39 countries; N = 99,075) and COVIDiSTRESS II (23 countries; N = 8293) projects, we examined whether country-level gender equality moderates the link between relationship status and perceived social support. Multilevel regression analyses indicated that gender equality moderated the link between relationship status and perceived social support. Single people in countries with less gender equality reported less perceived social support than was reported by both coupled people and single people from countries with moderate and high levels of gender equality, however, the effect of the interaction between relationship status and gender equality on perceived social support was very low. The results suggest that gender equality fosters perceived social support, both for single people and for partnered people.

## Introduction

Close and intimate relationships represent pivotal parts of human functioning as well as sources of social support that buffer against stress and loneliness^1,2^. Previous studies have shown that married individuals in intimate relationships report greater support from their significant others than single individuals^3–5^, and single individuals report lower general social support availability^6^. Moreover, although the benefits of romantic involvement are considered to be culturally universal^7^, the link between relationship status and perceived social support may be subjected to various factors operating not only at the individual level but also at the sociocultural level^6^.

One of the sociocultural factors that may moderate the link between relationship status and perceived social support is country-level gender equality, which includes, according to the World Economic Forum^8^, four critical dimensions: (1) economic participation and opportunity, (2) educational attainment, (3) health and survival, and (4) political empowerment. Past studies revealed that adolescents in countries with a high level of gender equality were more satisfied with their lives than adolescents in countries with a low level of gender equality were and that the level of social support explains the link between country-level gender equality and life satisfaction^9^. These results may be explained by the fact that in countries with greater gender equality, the division of labor in families is more egalitarian; thus, fathers are more likely to be actively involved in children's rearing, which results in a more supportive family climate^9^.

At the same time, countries with greater gender equality may adhere to values related to femininity^9^, such as modesty, cooperation, and support, which is in contrast to more “masculine” values, such as competition and domination^10–12^. These characteristics may explain the higher level of social support in countries with a high level of gender equality. According to Schwartz and Rubel-Lifschitz^12, p. 171]^, basic values are *broad, transsituational goals that vary in importance as guiding principles in life*. Considering that basic values, contrary to norms or attitudes, are not limited to specific actions or situations but permeate all aspects of our lives^12,13^, the link between gender equality and perceived social support should occur among adults and not only among adolescents. That suggests that, compared to the countries with a low level of gender equality, people in the countries with a high level of gender equality may have an easier access to the other sources of social support than a romantic relationship. Furthermore, previous studies suggest that well-being varies among single and coupled individuals across countries differing in the level of gender equality which seems to support this assumption. For instance, single people in the USA (Global Gender Gap Index = 0.789^8^) were found to be more satisfied with their life and to be subjectively more healthy than single people in Japan, which is less egalitarian (Global Gender Gap Index = 0.650^8^) than the USA is. This result can be explained by the greater level of familial support for American single people than for Japanese single people^14^.

Owing to the limited knowledge on the role of country-level gender equality in the link between relationship status and perceived social support across countries, in the present investigation, we explored whether the link between relationship status (i.e., not having a partner/spouse) and perceived social support among adult individuals varies as a function of country-level gender equality, which, in light of the past literature, appears to be an important factor contributing to a more supportive climate in societies^9^.

## The current study

The current investigation was intended to determine whether the link between relationship status (single vs. partnered) and perceived social support is moderated by country-level gender equality. We performed analyses on two large data samples (N = 99,075; 39 countries and N = 8293; 23 countries) collected by other authors in the scope of the COVIDiSTRESS I^15^ and COVIDiSTRESS II^16^ projects, which measured the functioning of people worldwide at the beginning of the COVID-19 pandemic (COVIDiSTRESS I) and a year after the pandemic (COVIDiSTRESS II). The data are available at https://osf.io/m5s8d and https://osf.io/wvsnk/ for COVIDiSTRESS I and COVIDiSTRESS II, respectively. Considering the links of perceived social support with age^17,18^, sex^3,17,18^, loneliness^3,19–21^ and perceived stress^20,22,23^, we included these variables in the analysis as covariates.

Drawing on previous studies presented in the introduction, we tested three hypotheses.

- H1. Country-level gender equality is positively related to perceived social support in such a way that higher country-level gender equality is related to greater social support perceived by individuals.
- H2. Relationship status is related to perceived social support in such a way that individuals in relationships report greater social support than single individuals.
- H3. The link between relationship status and perceived social support is moderated by country-level gender equality in such a way that the link between relationship status and perceived social support is stronger for countries with lower country-level gender equality.

## Methods

### Participants and procedure

The samples retrieved from the COVIDiSTRESS I and COVIDiSTRESS II datasets are convenience samples^15,16^. In both studies, the sole inclusion criteria were an age of at least 18 years and the provision of informed consent. In COVIDiSTRESS II, an attention check was also employed, and the duration of completion of the survey was controlled; that is, participants who did not pass the attention check and/or complete the survey in less than three minutes were excluded. The participants were recruited based on word of mouth, mailing lists, social media, and TV appeals. The participants did not receive any compensation, except for some Japanese participants of the COVIDiSTRESS I study, who were recruited from a crowd funding portal and obtained a symbolic reward equivalent to approximately 0.065 USD.

Given the nature of this study, we aimed to assess the measurement invariance of dependent variables; therefore, for the purpose of analyses, we included only the countries with at least 200 participants. Furthermore, we excluded participants from the countries for which data concerning the Global Gender Gap Index from 2022 were unavailable. Specifically, we excluded Croatia (N = 2965, COVIDiSTRESS I), Kosovo (N = 2707, COVIDiSTRESS I), and Taiwan (N = 2745, COVIDiSTRESS I and N = 221, COVIDiSTRESS II). Thus, we included 39 countries from the COVIDiSTRESS I database and 23 countries from the COVIDiSTRESS II database. Next, we excluded participants who declared that they were divorced or widowed and those who, regarding the question concerning relationship status, chose the option "dating" or "other or would rather not say" (12.70% and 21.4% for COVIDiSTRESS I and COVIDiSTRESS II, respectively) since these statuses do not directly indicate whether a person currently has a partner/spouse or not.

As a result of the above-indicated exclusions, our final sample consisted of 99,075 participants in the case of COVIDiSTRESS I and 8293 participants in the case of COVIDiSTRESS II. Regarding the sex distribution, the sample from COVIDiSTRESS I included 70,893 women (71.60%), 27,014 men (27.30%), and 1168 (1.20%) people who chose the option "other/rather not say" when answering the question concerning sex. The participants of COVIDiSTRESS I were 18–110 years old (*M* = 38.61, *SD* = 13.59). This sample comprised 61,991 partnered participants (62.60%) and 37,084 (37.40%) single participants. The sample from COVIDiSTRESS II included 5368 women (64.70%), 2867 men (34.60%), and 58 (0.70%) people who chose the option "other/rather not say" when answering the question concerning sex. The participants in COVIDiSTRESS II were between 18 and 88 years old (*M* = 39.74, *SD* = 14.04). This sample consisted of 5075 partnered participants (61.20%) and 3218 (38.80%) single participants. The detailed characteristics of the participants from each country are presented in Table 1.

**Table 1:** Characteristics of the samples.

Country	Individual-level variables (COVIDiSTRESS I)							Individual-level variables (COVIDiSTRESS II)						
	COVIDiSTRESS I Sample size (*n*)	Female (%)	Age *M (SD)*	Single status (%)	Loneliness *M (SD)*	Perceived social support *M (SD)*	Stress *M (SD)*	COVIDiSTRESS II Sample size (*n*)	Female (%)	Age *M (SD)*	Single status (%)	Loneliness *M (SD)*	Perceived social support *M (SD)*	Stress *M (SD)*
Argentina	4921	83.2	39.42 (14.71)	50.5	4.83 (3.08)	18.77 (4.55)	17.93 (7.83)	NA	NA	NA	NA	NA	NA	NA
Australia	266	75.9	41.71 (13.59)	28.2	5.05 (2.96)	19.92 (4.39)	16.31 (7.26)	NA	NA	NA	NA	NA	NA	NA
Austria	283	68.6	37.48 (11.00)	28.6	4.92 (2.91)	20.72 (3.69)	16.08 (7.23)	NA	NA	NA	NA	NA	NA	NA
Bangladesh	408	46.3	28.08 (6.11)	56.1	5.23 (2.57)	18.94 (4.13)	17.88 (6.16)	NA	NA	NA	NA	NA	NA	NA
Belgium	556	56.1	35.71 (12.34)	48.6	4.71 (3.04)	18.94 (4.40)	15.77 (7.32)	NA	NA	NA	NA	NA	NA	NA
Bosnia and Hercegovina	1064	73.0	36.60 (11.33)	38.7	5.68 (2.81)	18.79 (4.31)	18.06 (6.89)	NA	NA	NA	NA	NA	NA	NA
Brazil	652	71.6	33.65 (12.86)	59.4	5.23 (2.72)	20.47 (4.07)	20.75 (7.17)	336	70.8	38.99 (12.77)	38.4	5.38 (3.47)	13.11 (3.80)	20.78 (7.32)
Bulgaria	3884	79.8	39.79 (12.99)	32.6	5.09 (3.03)	19.09 (4.29)	18.16 (7.26)	205	68.3	42.31 (15.01)	25.4	4.12 (3.37)	11.63 (4.66)	17.91 (7.73)
Canada	413	65.1	39.87 (13.57)	35.4	5.09 (2.90)	19.15 (4.40)	17.32 (7.20)	NA	NA	NA	NA	NA	NA	NA
Columbia	NA	NA	NA	NA	NA	NA	NA	421	67.0	40.35 (12.26)	38.5	4.72 (3.37)	11.86 (4.82)	19.08 (6.87)
Costa Rica	NA	NA	NA	NA	NA	NA	NA	203	70.4	35.63 (9.82)	39.9	5.01 (3.25)	13.07 (4.10)	19.33 (7.24)
Czech Republic	1798	78.3	31.95 (10.18)	43.9	5.84 (2.90)	19.52 (4.17)	16.88 (7.11)	289	69.2	34.63 (10.96)	28.0	5.57 (3.18)	12.33 (4.73)	19.60 (6.66)
Denmark	9828	77.9	41.61 (13.91)	25.9	3.88 (2.64)	20.94 (3.74)	14.20 (7.15)	NA	NA	NA	NA	NA	NA	NA
Ecuador	NA	NA	NA	NA	NA	NA	NA	219	67.6	31.92 (10.80)	54.3	4.87 (3.18)	12.10 (4.85)	19.36 (6.30)
Estonia	NA	NA	NA	NA	NA	NA	NA	208	85.6	39.03 (9.50)	22.1	4.05 (3.22)	10.85 (5.39)	16.16 (6.97)
Finland	19,193	80.7	42.15 (13.65)	24.4	4.85 (3.04)	20.17 (4.17)	14.41 (7.36)	796	78.4	45.45 (13.84)	23.7	4.53 (3.23)	12.73 (4.48)	14.26 (7.08)
France	12,298	49.8	32.56 (12.07)	54.2	4.20 (3.06)	19.16 (4.35)	15.53 (7.39)	NA	NA	NA	NA	NA	NA	NA
Germany	1308	67.9	36.08 (11.66)	39.4	5.08 (2.98)	20.21 (4.03)	16.12 (6.92)	NA	NA	NA	NA	NA	NA	NA
Greece	436	78.2	44.84 (10.22)	75.5	4.18 (2.67)	20.07 (3.66)	16.39 (6.78)	NA	NA	NA	NA	NA	NA	NA
Guatemala	NA	NA	NA	NA	NA	NA	NA	219	82.6	37.79 (13.64)	35.2	4.39 (3.13)	13.09 (4.42)	18.92 (5.99)
Honduras	NA	NA	NA	NA	NA	NA	NA	284	68.0	26.32 (8.23)	69.0	5.21 (3.10)	8.91 (5.47)	19.58 (5.60)
Hungary	1167	68.5	47.16 (14.83)	22.1	5.33 (2.59)	19.24 (4.32)	17.27 (6.03)	NA	NA	NA	NA	NA	NA	NA
Indonesia	1497	66.8	30.77 (9.24)	50.2	4.02 (2.84)	17.95 (3.92)	17.39 (5.90)	NA	NA	NA	NA	NA	NA	NA
Ireland	195	80.0	40.01 (10.36)	25.1	4.69 (2.81)	20.04 (3.89)	15.03 (6.97)	278	64.0	30.03 (11.10)	63.7	6.53 (3.56)	11.59 (4.71)	21.02 (7.29)
Italy	1404	74.3	44.36 (14.80)	37.5	5.09 (2.85)	19.24 (3.95)	15.05 (6.79)	214	72.0	46.15 (15.51)	35.0	4.78 (3.22)	10.85 (4.73)	18.89 (7.13)
Japan	4536	43.8	44.01 (11.33)	40.9	4.31 (2.66)	12.68 (5.09)	20.17 (5.73)	1848	39.4	45.56 (10.96)	40.7	4.61 (3.12)	7.94 (4.50)	18.42 (6.02)
Korea, South	458	46.7	37.79 (10.19)	46.7	4.22 (2.63)	18.59 (4.00)	16.97 (6.71)	NA	NA	NA	NA	NA	NA	NA
Kyrgyzstan	NA	NA	NA	NA	NA	NA	NA	191	82.7	33.60 (12.07)	37.7	3.82 (2.85)	11.30 (4.61)	17.66 (5.98)
Lithuania	7126	73.8	37.62 (11.90)	24.1	4.68 (2.85)	19.55 (3.77)	14.93 (6.85)	NA	NA	NA	NA	NA	NA	NA
Malaysia	532	74.1	36.32 (14.27)	56.6	4.32 (2.93)	18.55 (4.21)	17.16 (7.08)	185	70.3	27.43 (9.12)	78.9	6.59 (3.61)	10.65 (4.64)	22.15 (6.33)
Mexico	8264	72.5	36.19 (13.26)	56.0	4.48 (3.05)	20.32 (4.34)	17.33 (7.37)	NA	NA	NA	NA	NA	NA	NA
Netherlands	1271	73.7	44.07 (14.43)	26.5	4.41 (2.66)	20.11 (3.63)	13.09 (6.78)	NA	NA	NA	NA	NA	NA	NA
Norway	NA	NA	NA	NA	NA	NA	NA	308	79.9	40.90 (13.49)	17.9	5.10 (3.41)	12.95 (4.40)	16.55 (7.50)
Pakistan	349	67.6	27.06 (8.83)	67.9	5.00 (3.08)	18.42 (4.38)	18.03 (7.32)	NA	NA	NA	NA	NA	NA	NA
Panama	686	74.5	38.55 (14.45)	47.7	3.58 (2.49)	20.80 (3.84)	14.18 (6.32)	NA	NA	NA	NA	NA	NA	NA
Philippines	526	67.7	25.43 (10.95)	79.3	5.27 (2.69)	18.31 (4.51)	20.53 (6.39)	NA	NA	NA	NA	NA	NA	NA
Poland	2885	87.1	31.29 (7.55)	23.9	6.11 (3.12)	19.55 (3.95)	19.85 (7.22)	NA	NA	NA	NA	NA	NA	NA
Portugal	957	84.6	32.38 (12.71)	60.0	4.77 (2.81)	20.22 (3.84)	18.90 (7.26)	349	69.1	34.53 (15.33)	61.9	5.08 (3.40)	12.32 (4.42)	20.11 (7.53)
Romania	257	72.8	34.26 (9.48)	27.6	5.57 (2.66)	19.12 (4.14)	16.53 (6.58)	NA	NA	NA	NA	NA	NA	NA
Serbia	235	66.0	38.11 (12.53)	41.3	5.42 (2.73)	19.58 (3.96)	16.74 (6.50)	NA	NA	NA	NA	NA	NA	NA
Slovakia	786	74.3	40.34 (12.30)	28.6	5.74 (2.83)	19.24 (4.34)	16.59 (6.90)	211	88.2	35.08 (13.09)	39.3	5.82 (3.03)	11.77 (4.66)	19.99 (6.46)
Spain	534	69.5	37.22 (14.61)	42.5	4.49 (2.97)	19.66 (4.44)	16.57 (7.24)	445	63.8	41.44 (13.26)	32.8	4.32 (3.06)	13.22 (4.20)	18.79 (6.82)
Sweden	2580	74.7	45.93 (12.19)	20.1	4.60 (2.93)	20.50 (3.91)	14.46 (6.86)	NA	NA	NA	NA	NA	NA	NA
Switzerland	1044	59.3	41.09 (16.70)	44.8	4.38 (2.82)	20.44 (3.92)	13.85 (6.47)	527	62.4	44.31 (18.75)	24.1	3.48 (2.86)	13.27 (3.91)	14.09 (6.48)
Turkey	1096	74.8	32.63 (11.08)	56.3	5.30 (2.34)	19.56 (4.41)	21.27 (6.85)	144	66.7	23.74 (7.65)	84.7	6.26 (3.31)	11.13 (4.29)	24.65 (6.43)
Ukraine	NA	NA	NA	NA	NA	NA	NA	198	63.1	32.06 (10.05)	35.4	5.76 (3.56)	12.55 (4.48)	18.90 (7.12)
United Kingdom	1322	76.0	38.39 (12.29)	29.8	5.02 (2.95)	19.84 (3.94)	17.20 (7.52)	NA	NA	NA	NA	NA	NA	NA
United States	2060	75.1	41.54 (14.24)	33.9	4.95 (3.01)	20.39 (3.99)	17.45 (7.45)	NA	NA	NA	NA	NA	NA	NA
Uruguay	NA	NA	NA	NA	NA	NA	NA	215	86.0	41.67 (12.07)	20.5	4.00 (3.20)	14.08 (4.20)	16.76 (6.72)

### Measures and instruments

The instruments used in the COVIDiSTRESS I and COVIDiSTRESS II survey,

were translated from English into 47 languages as described by Yamada et al.^15^. After the direct translations, the back-translations to English were completed, and the final versions of the instruments were finalized through panel discussions.

#### Individual-level measures

*Relationship status.* Relationship status in COVIDiSTRESS I was assessed by the indication of belonging to one of three categories or marital status: "1 = single, 2 = married/cohabiting, 3 = divorced/widowed", with the possibility of choosing the option "4 = other or would rather not say". In COVIDiSTRESS II, "cohabitating" and "dating" were separate options. In the present analyses, people who chose "single" were classified as single. People who chose "married" or "cohabitating" were classified as partnered. People who chose other options were excluded from the current analyses.

*Perceived social support.* Perceived social support for COVIDiSTRESS I was assessed by the Social Provisions Scale—Short Form (SPS-10;^24^). However, assuming that the anticipated effect size would be 0.3 and to ensure sufficient statistical power to assess the measurement invariance across countries^25^, we decided to use the 5-item version of the original SPS-10 that was developed by Orpana et al.^26^. Five items (e.g., "I have close relationships that provide me with a sense of emotional security and well-being") were rated using a 6-point Likert scale ranging from 0 (*strongly disagree*) to 5 (*strongly agree*). The Cronbach’s alpha for this scale ranges from 0.79 (France) to 0.88. (Australia and Japan). Perceived social support for COVIDiSTRESS II was assessed with the Perceived Support Scale^27^. This scale consists of three items (e.g., “People would help me if I needed it”) that are rated on a 7-point Likert scale ranging from 0 (*strongly disagree*) to 6 (*strongly agree*). The Cronbach’s alpha for this scale ranges from 0.71 (Costa Rica and Switzerland) to 0.85 (Estonia).

*Loneliness.* Loneliness was measured by the use of the Three-Item Loneliness Scale^28^. Three items (e.g., “How often do you feel that you lack companionship?”) were rated on a 5-point Likert scale from 0 (*never*) to 4 (*very often*). The Cronbach’s alpha for this scale ranges from 0.70 (Pakistan, COVIDiSTRESS I) to 0.93 (Japan, COVIDiSTRESS II). According to COVIDiSTRESS I, the alpha for five countries (Bangladesh, Greece, Panama, Serbia, and Turkey) was less than 0.70. Thus, we excluded them from the regression models for loneliness in COVIDiSTRESS I.

*Perceived stress.* Perceived stress was assessed with the Perceived Stress Scale (PSS-10;^29^) for COVIDiSTRESS I and COVIDiSTRESS II. The scale has two factors, "negative" and "positive," and consists of reverse-coded items. The items of the negative factor assess the frequency of six distress-related experiences (e.g., "Felt difficulties were piling up so high that you could not overcome them"). The items of the positive factor assess the frequency of four coping-related experiences (e.g., "being able to control irritations in your life"). The participants assessed the frequency of these experiences during the week before the study using a scale ranging from 0 (*never*) to 4 (*very often*). The Cronbach's alpha for this scale ranges from 0.79 (Japan, COVIDiSTRESS I) to 0.91 (Estonia, Norway, and Portugal, COVIDiSTRESS II).

#### Country-level measures

*Gender equality****.*** Country-level gender equality was assessed by employing the Global Gender Gap Index^8^ since it includes not only the measures captured by the Gender Empowerment Measure (GEM), Gender Development Index (GDI), or Gender Inequality Index (GII) but also the ratios of labor force participation; literacy rates; enrollment in primary, secondary, and tertiary education; sex ratios at birth; ratios of life expectancy; ratios of men to women in ministerial positions; and years with a male versus a female head of state. The index has a hypothetical range from *0* (absolute lack of gender equality) to *1* (perfect gender equality)^30^. However, the actual values ranged from 0.56 (Pakistan) to 0.86 (Finland) in our samples and from 0.51 (Yemen) to 0.86 (Finland) in the total ranking.

### Data analysis

In the first step of the analysis, we computed the descriptive statistics for loneliness, perceived social support, and perceived stress for each country.

In the second step, we assessed the measurement invariance analysis to check whether the research tools assessing dependent variables have the same structure in all countries^31^. We assessed the invariance with the criteria described by Hu and Bentler^32^, according to whom a comparative fit index (CFI) higher than 0.90 is an indicator of acceptable fit and a root-mean-square error of approximation (RMSEA) lower than 0.08 is an indicator of a lack of misfit.

For the most critical step, we conducted a multilevel analysis. We started with the zero model—a random-intercept-only model (without predictors). We calculated the intraclass correlation (ICC) to determine the proportion of variance in perceived social support explained by country-level clustering. The ICCs (intraclass coefficients) were 0.09 for perceived social support for COVIDiSTRESS I and COVIDiSTRESS II. These results indicate that the differences between countries accounted for 9% of the variance in perceived social support in both databases.

Next, we tested a model predicting the level of perceived social support from relationship status as the independent variable at the individual level; gender, age, loneliness, and perceived stress as individual-level covariates; HDI as a country-level covariate; and the Global Gender Gap Index (GGGI) as a moderator. Individual-level variables were group-mean-centered, and country-level variables were grand-mean-centered^33^. Like Luchetti and colleagues^34^, the effects of the independent variables on the dependent variable were allowed to vary from country to country because of the nested data structure.

The analyses were conducted with R software (version 4.2.1). Analysis of invariance was conducted with the lavaan package^35^, and multilevel analyses were performed with the plm, lme4, lmerTest, and jtools packages^36–38^. The analysis R code is available in the online supplementary materials.

#### Preliminary analyses

Descriptive statistics for each country has been presented in Table 1.

In both samples, the primary study variables were correlated with each other (see Table 2).

**Table 2:** Correlations between the Major Study Variables.

	COVIDiSTRESS I						COVIDiSTRESS II				
	1	2	3	4	5		1	2	3	4	5
1. Loneliness	–					1. Loneliness	–				
2. Perceived social support	− 0.24**	–				2. Perceived social support	− 0.32**	–			
3. Perceived stress	0.57**	− 0.28**	–			3. Perceived stress	0.59**	− 0.30**	–		
4. Age	− 0.21**	0.02**	− 0.27**	–		4. Age	− 0.24**	− 0.02*	− 0.29**	–	
5. Gender equality	− 0.01**	0.22**	− 0.20**	0.08**	–	5. Gender equality	− 0.03*	.29**	− 0.18**	0.02*	–

*p < .05; **p < .01.

As Table 2 shows, in both samples, country-level gender equality was positively correlated with perceived social support and negatively correlated with perceived stress and loneliness. In both samples, age was negatively correlated with perceived stress and loneliness. For COVIDiSTRESS I, age was positively correlated with perceived social support, whereas for COVIDiSTRESS II, the correlation between age and perceived social support was negative. However, the correlations between age and perceived social support were very low in both samples.

In addition, Table 3 presents the results of the comparisons between single and partnered individuals regarding perceived stress, loneliness, and perceived social support.

**Table 3:** Differences between single and partnered individuals—unpaired T-Tests.

	COVIDiSTRESS I					COVIDiSTRESS II				
	Single Individuals		Partnered Individuals		*t*	Single Individuals		Partnered Individuals		*t*
	*M*	*SD*	*M*	*SD*		*M*	*SD*	*M*	*SD*	
Perceived Stress	17.55	7.60	15.30	7.13	44.476**	20.03	7.05	17.12	6.79	18.751**
Loneliness	5.32	3.09	4.27	2.82	51.615**	5.90	3.37	4.09	3.04	24.742**
Perceived social support	18.33	5.01	20.11	4.08	− 50.440**	10.33	5.06	11.89	4.77	− 13.941**

**p < .001.

As Table 3 shows, single participants reported greater perceived stress and loneliness and less perceived social support than did partnered individuals in both samples.

Finally, the primary analyses were preceded by the assessment of the measurement invariance of perceived stress, loneliness, and perceived social support across countries by conducting the CFA analysis^31^. The detailed results concerning the measurement invariance analyses are reported in Table S1 in the online supplementary materials. The analyses demonstrated that all the measures met the criteria of configural invariance. All the measures except for the Three-Item Loneliness Scale in the COVIDiSTRESS I sample met the criterion of metric invariance. The three-item Loneliness Scale in the COVIDiSTRESS I sample had a slightly greater RMSEA than needed. This may be due to the large sample size^39^. Thus, the measurement invariance of the tools used to measure dependent variables in our samples is acceptable.

#### Relationship status and country-level gender equality and perceived social support

Table 4 presents the model predicting perceived social support from relationship status and country-level gender equality while controlling for covariates.

**Table 4:** Predictors of perceived social support.

	COVIDiSTRESS I														COVIDiSTRESS II													
	Model 2							Model 3 (with interactions)							Model 2							Model 3 (with interactions)						
	B	SE	beta	*p*	f2	f2 (CI 90%)		B	SE	beta	*p*	f2	f2 (CI 90%)		B	SE	beta	*p*	f2	f2 (CI 90%)		B	SE	beta	*p*	f2	f2 (CI 90%)	
(Intercept)	19.76	0.23	0.00	< .001				19.76	0.23	0	< .001				11.75	0.30	0.00	< .001				11.75	0.30		< .001			
Relationship status	− 1.48	0.03	− 0.15	< .001	0.03	0.03	0.03	− 1.49	0.03	− 0.15	< .001	0.03	0.03	0.03	− 0.64	0.11	− 0.06	< .001	0.00	0.00	0.01	− 0.62	0.11	− 0.06	< .001	0.00	0.00	0.01
Sex	1.39	0.03	0.13	< .001	0.02	0.02	0.03	1.38	0.03	0.13	< .001	0.02	0.02	0.03	1.15	0.10	0.11	< .001	0.01	0.01	0.02	1.15	0.10	0.10	< .001	0.01	0.01	0.02
Age	− 0.03	0.00	− 0.10	< .001	0.01	0.01	0.01	− 0.03	0.00	− 0.09	< .001	0.01	0.01	0.01	− 0.03	0.00	− 0.08	< .001	0.01	0.00	0.01	− 0.03	0.00	− 0.08	< .001	0.01	0.00	0.01
Loneliness	− 0.23	0.01	− 0.15	< .001	0.02	0.02	0.02	− 0.23	0.01	− 0.15	< .001	0.02	0.02	0.02	− 0.36	0.02	− 0.23	< .001	0.05	0.04	0.06	− 0.36	0.02	− 0.17	< .001	0.05	0.04	0.06
Stress	− 0.11	0.00	− 0.17	< .001	0.03	0.02	0.03	− 0.11	0.00	− 0.17	< .001	0.03	0.02	0.03	− 0.13	0.01	− 0.17	< .001	0.03	0.02	0.03	− 0.13	0.01	− 0.23	< .001	0.03	0.02	0.03
HDI	− 1.43	2.59	− 0.02	0.58	0.01	0.00	0.13	− 1.45	2.59	− 0.02	0.58	0.01	0.00	0.13	− 3.19	3.34	− 0.06	0.35	0.04	0.00	0.34	− 3.19	3.34	− 0.06	0.35	0.04	0.00	0.34
GGGI	12.38	4.27	0.16	0.01	0.24	0.04	0.59	12.37	4.27	0.16	0.01	0.24	0.04	0.59	14.19	5.69	0.20	0.02	0.31	0.02	0.90	14.20	5.69	0.20	0.02	0.31	0.02	0.90
GGGI x relationship status								− 3.09	0.53	− 0.02	< .001	0.00	0.00	0.00								3.13	1.47	0.02	0.03	0.00	0.00	0.00

Relationship status: 0 = partnered; 1 = single. Sex: 0 = man; 1 = woman. GGGI = Global Gender Gap Index.

**Figure 1:**
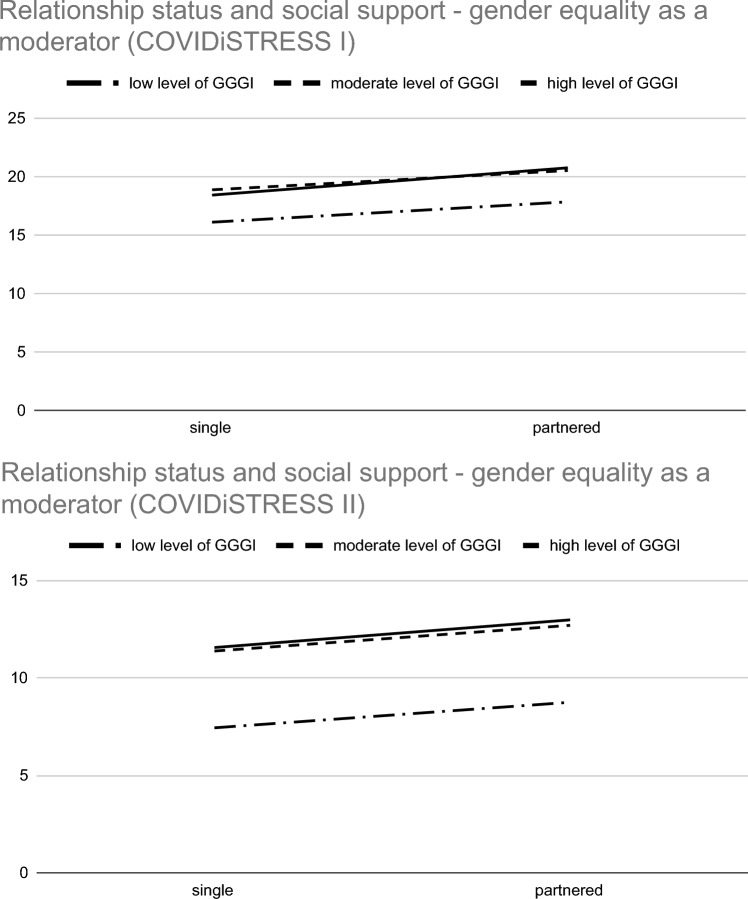
Relationship status and perceived social support—country-level gender equality as a moderator.

As Table 4 shows, in both samples, country-level gender equality was positively associated with perceived social support, and the Cohen f2 effects for this association can be interpreted as medium^40^. Additionally, the link between relationship status and perceived social support was moderated by country-level gender equality, although the effects of this link were close to zero (see Fig. 1).

Like Molina and Simon^41^, we decided to check whether the differences between the slopes were significant (see Table S2 and S3 in the online supplementary materials). We used the significance of the difference between two slopes calculation tool^42^. We applied the Holm correction for multiple comparisons^43^. For COVIDiSTRESS I, the difference between slopes for the countries with low and high levels of gender equality was significant (*p* < .001). The difference between slopes for the countries with moderate and high levels of gender equality was also significant (*p* < .001). This finding suggested that in the countries with the highest level of gender equality, the difference between single and partnered people was the greatest. The difference between slopes for the countries with low and moderate levels of gender equality was marginally significant (*p* = .054). For COVIDiSTRESS II, the difference between slopes for the countries with low and moderate levels of gender equality was significant (*p* < .001). The difference between the slopes for the countries with low and high levels of gender equality was also significant (*p* < .001). These findings suggest that contrary to the results of COVIDiSTRESS I, for COVIDiSTRESS II, in the countries with the lowest level of gender equality, the difference between single and partnered people was the greatest.

## Discussion

The current investigation aimed to determine whether country-level gender equality predicts the level of perceived social support and whether it may play a moderating role in the link between relationship status and perceived social support. First, the findings showed that country-level gender equality positively predicts perceived social support, which is consistent with the findings of previous studies^9^ and with H1. The findings supported H2 and past studies^3–6^ by demonstrating that being in a romantic relationship is related to greater perceived social support.

Second, our findings provide insight not only into the link between country-level gender equality and perceived social support in general but also into country-level gender equality as a moderator of the link between relationship status and perceived social support. H3 assumed that the link between relationship status and perceived social support would be stronger for countries with a low level of country-level gender equality and was partially supported. Contrary to our predictions, for COVIDiSTRESS I, the link between relationship status and perceived social support was the strongest for countries with a high level of gender equality. Schobin^11^ demonstrated that the link between relationship status and loneliness is also the strongest for countries with a high level of gender equality. Schobin^11^ explains this finding with the assumption that in egalitarian countries, people may be more inclined to end a low-quality relationship than to continue it at all costs; thus, the difference between single and partnered people's well-being increases. Our data analysis revealed that a similar phenomenon might occur for COVIDiSTRESS I. However, for COVIDiSTRESS II, the link between relationship status and perceived social support was the strongest for countries with a low level of country-level gender equality, which was consistent with H3.

Furthermore, along with an assessment of the significance of the interactional effects, we estimated, in line with other researchers' recommendations (e.g.,^44^), the effect size of the observed differences, which were nearly zero. The magnitude of effect being zero implied that, contrary to our hypothesis, the link between relationship status and perceived social support was not moderated by the level of gender equality across countries; we consider these findings to have substantive significance that are relevant to specific contexts and situations^44^. In other words, even though we demonstrated very little evidence of mean-level perceived social support as a function of gender equality, significant individual differences in perceived social support levels could still exist. This means that some single and coupled individuals might have experienced different levels of perceived social support across countries that vary in gender equality. Moreover, based on previous cross-country studies analyzing loneliness (e.g.,^45^), we can also suggest that even slight differences in perceived social support (resulting, for example, in loneliness) as a function of country differences in gender equality may have cumulative effects because weak changes (e.g., increased loneliness) tend to represent risk factors for adverse outcomes in health and well-being domains^45,46^.

Finally, our analyses also demonstrated that these differences are a function of countries that include non-WEIRD countries (i.e., which are not Western, Educated, Industrialized, Rich, and Democratic) that are severely underrepresented in the social sciences^47^. Our findings indicated a positive association between perceived social support and country-level gender equality in COVIDiSTRESS I and COVIDiSTRESS II. This finding is consistent with past studies that have shown the importance of gender equality for various life outcomes, such as somatic health^48,49^, depression^50,51^, and life satisfaction^9,52^.

Finally, while some might consider gender equality a woman or girl issue (e.g.,^9^, p. 1083), this investigation reveals that single women and single men benefit from greater societal gender equality. The association between societal gender equality and perceived social support among single individuals reveals that single individuals as a group may be subjected to negative stereotyping and discrimination due to the lack of a romantic partner/spouse^6,53^ and are at risk of lower social support^3,5,6^. Individuals in these countries may benefit from greater gender equality, which fosters a more socially supportive climate^9^. In other words, our results imply that gender equality may act as a buffer against low perceived social support among single women and men.

The current investigation should be interpreted in light of several limitations. First, the samples enrolled in COVIDiSTRESS I and COVIDiSTRESS II were convenience samples. Therefore, these data may not be representative of each country. We need to replicate the analyses of the data collected from more representative country samples to obtain more accurate results. Second, the COVIDiSTRESS I data were collected at the beginning of the COVID-19 pandemic outbreak, whereas the COVIDiSTRESS II data were collected one year later.

Third, in the COVIDiSTRESS I and COVIDiSTRESS II projects, perceived social support was operationalized differently. Specifically, the Social Provisions Scale-5^25^, used in COVIDiSTRESS I, contains items referring mainly to access to social provisions in general. In contrast, the Perceived Support Scale^26^, used in COVIDiSTRESS II, contains items referring to access to help during crises.

Fourth, there are different kinds of social support—informational, emotional, and tangible^54^. Furthermore, according to Melrose and colleagues^55^, studies on social support should also take into account the distinction between received social support, which denotes the number of supportive behaviors toward an individual, and perceived social support, which denotes not only the quantity of supportive behaviors but also the satisfaction of the individual with the behaviors. Thus, in future studies, a more nuanced approach to social support could provide a deeper understanding of its link with relationship status and country-level gender equality.

Finally, the data collected from the COVIDiSTRESS I and COVIDiSTRESS II samples did not include an assessment of the quality of the relationship. There is evidence that the beneficial effects of a romantic relationship on quality of life occur only when the quality of the relationship is high^56–58^. Thus, future studies could benefit from controlling for the quality of relationships.

### Supplementary Information

Supplementary Information.

## Data Availability

The datasets generated and/or analyzed during the current study are available in the OSF repository at https://osf.io/m5s8d and https://osf.io/wvsnk/ for COVIDiSTRESS I and COVIDiSTRESS II, respectively.
