# Supplementary material for: Exploring the role of country-level gender equality in the link between relationship status and perceived social support across 49 countries
Source: Sci Rep. 2024 Jan 29;14:2436. doi: 10.1038/s41598-024-52304-z (PMC10825160; doi:10.1038/s41598-024-52304-z)
Supplement: Supplementary file 1 — Supplementary Information. [file 41598_2024_52304_MOESM1_ESM.docx]

**Analysis of invariance - the R code**

library(lavaan)

library(haven)

library(semTools)

library(semPlot)

library(Rcpp)

library(plm)

library(REndo)

library(lme4)

library(ggplot2)

library(HLMdiag)

library(DHARMa)

library(car)

library(tidyverse)

library(dplyr)

library(RColorBrewer)

library(lmerTest)

library(sjstats)

library(olsrr)

library(jtools)

library(moments)

library(lmtest)

library(ltm)

library(psych)

library(dgof)

library(Hmisc)

#multilevel CFA: support COVIDiSTRESS I

covidistress <- read_sav("covidistress.sav")

sps5=data.frame(covidistress$country,covidistress$SPS_4,covidistress$SPS_5,covidistress$SPS_6,covidistress$SPS_3, covidistress$SPS_10)

sps<-na.omit(sps5)

View(sps)

#overall model first

sps.scale<-'F1=~covidistress.SPS_3+covidistress.SPS_4+covidistress.SPS_5+covidistress.SPS_6+covidistress.SPS_10'

bootstrap=1000L

sps.fit<-cfa(sps.scale,data=sps,estimator="MLR", meanstructure=TRUE)

summary(sps.fit,fit.measures=TRUE,rsq=TRUE,standardized=TRUE)

#type of variables

typeof(sps$covidistress.country)

typeof(sps$covidistress.SPS_3)

sps$covidistress.country <- as.numeric(sps$covidistress.country)

sps$covidistress.SPS_3 <- as.numeric(sps$covidistress.SPS_3)

sps$covidistress.SPS_4 <- as.numeric(sps$covidistress.SPS_4)

sps$covidistress.SPS_5 <- as.numeric(sps$covidistress.SPS_5)

sps$covidistress.SPS_6 <- as.numeric(sps$covidistress.SPS_6)

sps$covidistress.SPS_10 <- as.numeric(sps$covidistress.SPS_10)

#configural invariance

config<-cfa(sps.scale,data=sps,estimator="MLR", group="covidistress.country")

summary(config,fit.measures=TRUE,rsq=TRUE,standardized=TRUE)

#weak

weak<-cfa(sps.scale,data=sps,estimator="MLR",group="covidistress.country", group.equal="loadings")

summary(weak,fit.measures=TRUE,rsq=TRUE,standardized=TRUE)

#strong

strong<-cfa(sps.scale,data=sps,estimator="MLR",group="covidistress.country", group.equal=c("loadings", "intercepts"))

summary(strong,fit.measures=TRUE,rsq=TRUE,standardized=TRUE)

#strict

strict<-cfa(sps.scale,data=sps,estimator="MLR",group="covidistress.country", group.equal=c("loadings", "intercepts", "residuals"))

summary(strict,fit.measures=TRUE,rsq=TRUE,standardized=TRUE)

#anova

anova(config, weak, strong, strict)

#invariance

measurementInvariance(model=sps.scale,

data=sps,

group="covidistress.country")

#multilevel CFA: support COVIDiSTRESS II

covidistress2 <- read_sav("covidistress2.sav")

support=data.frame(covidistress2$country,covidistress2$perceived_support_1_midneutral,covidistress2$perceived_support_2_midneutral,covidistress2$perceived_support_3_midneutral)

sup<-na.omit(support)

View(sup)

#overall model first

sup.scale<-'F1=~covidistress2.perceived_support_1_midneutral+covidistress2.perceived_support_2_midneutral+covidistress2.perceived_support_3_midneutral'

bootstrap=1000L

sup.fit<-cfa(sup.scale,data=sup,estimator="MLR", meanstructure=TRUE)

summary(sup.fit,fit.measures=TRUE,rsq=TRUE,standardized=TRUE)

#type of variables

typeof(sup$covidistress2.country)

typeof(sup$covidistress2.perceived_support_1_midneutral)

sup$covidistress2.country <- as.numeric(sup$covidistress2.country)

sup$covidistress2.perceived_support_1_midneutral <- as.numeric(sup$covidistress2.perceived_support_1_midneutral)

sup$covidistress2.perceived_support_2_midneutral <- as.numeric(sup$covidistress2.perceived_support_2_midneutral)

sup$covidistress2.perceived_support_3_midneutral <- as.numeric(sup$covidistress2.perceived_support_3_midneutral)

#configural invariance

config<-cfa(sup.scale,data=sup,estimator="MLR", group="covidistress2.country")

summary(config,fit.measures=TRUE,rsq=TRUE,standardized=TRUE)

#weak

weak<-cfa(sup.scale,data=sup,estimator="MLR",group="covidistress2.country", group.equal="loadings")

summary(weak,fit.measures=TRUE,rsq=TRUE,standardized=TRUE)

#strong

strong<-cfa(sup.scale,data=sup,estimator="MLR",group="covidistress2.country", group.equal=c("loadings", "intercepts"))

summary(strong,fit.measures=TRUE,rsq=TRUE,standardized=TRUE)

#strict

strict<-cfa(sup.scale,data=sup,estimator="MLR",group="covidistress2.country", group.equal=c("loadings", "intercepts", "residuals"))

summary(strict,fit.measures=TRUE,rsq=TRUE,standardized=TRUE)

#anova

anova(config, weak, strong, strict)

#invariance

measurementInvariance(model=sup.scale,

data=sup,

group="covidistress2.country")

**Analysis of invariance - results**

**Table S1 - invariance**

|  | CFI | RMSEA | CFI delta | RMSEA delta |
| --- | --- | --- | --- | --- |
|  | | | | |
| Social Provision Scale-5 (COVIDiSTRESS I) | | | | |
| configural | .98 | .08 | NA | |
| loadings | .97 | .08 | .01 | .01 |
| intercepts | .94 | .09 | .03 | .02 |
| means | .90 | .12 | .04 | .03 |
| Perceived Support Scale (COVIDiSTRESS II) | | | | |
| configural | 1.00 | .00 | NA | |
| loadings | .99 | .06 | .01 | .06 |
| intercepts | .96 | .10 | .03 | .04 |
| means | .76 | .21 | .20 | .12 |

**Multilevel analysis - the R code**

library(lavaan)

library(haven)

library(semTools)

library(semPlot)

library(Rcpp)

library(plm)

library(REndo)

library(lme4)

library(ggplot2)

library(HLMdiag)

library(DHARMa)

library(car)

library(tidyverse)

library(dplyr)

library(RColorBrewer)

library(lmerTest)

library(sjstats)

library(olsrr)

library(jtools)

library(moments)

library(lmtest)

library(ltm)

library(psych)

library(dgof)

library(Hmisc)

covidistress <- read_sav("covidistress.sav")

#social support model

model1 <- lmer(formula=sup_sum~ 1+single_c+gender_c+age_c+pss_c+slon_c+hdi_c+(1|country),

data=covidistress_lon)

summ(model1)

confint(model1)

stdCoef.merMod <- function(object) {

sdy <- sd(getME(object,"y"))

sdx <- apply(getME(object,"X"), 2, sd)

sc <- fixef(object)*sdx/sdy

se.fixef <- coef(summary(object))[,"Std. Error"]

se <- se.fixef*sdx/sdy

return(data.frame(stdcoef=sc, stdse=se))

}

stdCoef.merMod(model1)

#second level predictor - GGGI

#model without moderation

model2 <- lmer(sup_sum~1+single_c+gender_c+age_c+pss_c+slon_c+hdi_c+gggi_c+(1|country), data=covidistress_lon)

summ(model2)

confint(model2)

stdCoef.merMod(model2)

#model with moderation

model3<-lmer(sup_sum~single_c+gender_c+age_c+pss_c+slon_c+hdi_c+gggi_c+single_c*gggi_c+(1|country),data=covidistress_lon)

summ(model3)

confint(model3)

stdCoef.merMod(model3)

**The differences between slopes**

**Table S2. Slopes - support**

|  |  | COVIDiSTRESS I | | | COVIDiSTRESS II | | |
| --- | --- | --- | --- | --- | --- | --- | --- |
| low GGGI (below 1 SD) | variable | estimate | SE | df | estimate | SE | df |
|  | intercept | 18.68** | 0.48 | 14.27 | 9.50* | 1.13 | 2.00 |
|  | relationship status (single = 1) | -1.52** | 0.09 | 12646.36 | -1.40** | 0.21 | 1966 |
|  | gender (woman = 1) | 1.36** | 0.09 | 12646.43 | 1.10** | 0.20 | 1966 |
|  | age | -0.04** | 0.01 | 12646.34 | -0.04** | 0.01 | 1966 |
|  | stress | -0.12** | 0.01 | 12646.31 | -0.17** | 0.02 | 1966 |
|  | loneliness | -0.20** | 0.02 | 12646.32 | -0.20** | 0.04 | 1966 |
| moderate GGGI (+/- 1 SD) | intercept | 19.80** | 0.14 | 24.282 | 12.07** | 0.28 | 18.04 |
|  | relationship status (single = 1) | -1.33** | 0.04 | 46348.24 | -0.37* | 0.15 | 4858.10 |
|  | gender (woman = 1) | 1.24** | 0.04 | 46348.39 | 1.14** | 0.14 | 4858.06 |
|  | age | -0.03** | 0.01 | 46349.26 | -0.03** | 0.01 | 4858.07 |
|  | stress | -0.11** | 0.01 | 46348.04 | -0.11** | 0.01 | 4858.08 |
|  | loneliness | -0.22** | 0.02 | 46348.98 | -0.38** | 0.02 | 4858.06 |
| high GGGI (above 1 SD) | intercept | 20.17** | 0.03 | 15502 | 12.45** | 0.34 | 2.82 |
|  | relationship status (single = 1) | -1.94** | 0.07 | 15502 | -0.21 | 0.26 | 1361 |
|  | gender (woman = 1) | 1.58** | 0.08 | 15502 | 1.27** | 0.25 | 1361 |
|  | age | -0.04** | 0.01 | 15502 | --0.04** | 0.01 | 1361 |
|  | stress | -0.10** | 0.01 | 15502 | -0.12** | 0.02 | 1361 |
|  | loneliness | -0.28** | 0.01 | 15502 | -0.48** | 0.04 | 1361 |

* p < .05 ** p < .001

**Table S3. Social support - differences between slopes**

|  | COVIDiSTRESS I | | | COVIDiSTRESS II | | |
| --- | --- | --- | --- | --- | --- | --- |
|  | t | df | p | t | df | p |
| low vs. moderate | 1.93 | 79.88 | .054 | 3.99 | 6.91 | <.001* |
| low vs. high | 3.68 | 36.32 | <.001* | 3.56 | 3.37 | <.001* |
| moderate vs. high | 7.57 | 81.94 | <.001* | 0.53 | 6.30 | 0.594 |

*the difference is significant after Holm correction (assuming the p = .05 as a threshold)
